# Classification algorithm for congenital Zika Syndrome: characterizations, diagnosis and validation

**DOI:** 10.1038/s41598-021-86361-5

**Published:** 2021-03-24

**Authors:** Rafael V. Veiga, Lavinia Schuler-Faccini, Giovanny V. A. França, Roberto F. S. Andrade, Maria Glória Teixeira, Larissa C. Costa, Enny S. Paixão, Maria da Conceição N. Costa, Maurício L. Barreto, Juliane F. Oliveira, Wanderson K. Oliveira, Luciana L. Cardim, Moreno S. Rodrigues

**Affiliations:** 1 Center of Data and Knowledge Integration for Health (CIDACS), Instituto Gonçalo Moniz, Fundação Oswaldo Cruz, Salvador, Bahia Brazil; 2 Instituto de Ciências da Saúde, Universidade Federal da Bahia, Salvador, Bahia Brazil; 3 Universidade Federal do Rio Grande do Sul, Rio Grande do Sul, Brazil; 4 Secretariat of Health Surveillance, Ministry of Health, Brasilia, Brazil; 5 Instituto de Física, Universidade Federal da Bahia, Salvador, Bahia Brazil; 6 Instituto de Saúde Coletiva, Universidade Federal da Bahia, Salvador, Bahia Brazil; 7 London School of Hygiene and Tropical Medicine, London, England United Kingdom; 8 Department of Mathematics, Centre of Mathematics of the University of Porto (CMUP), Porto, Portugal; 9 Hospital das Forças Armadas, Ministério da Defesa, Distrito Federal, Brasília, Brazil; 10 Fundação Oswaldo Cruz, Porto Velho, Rondônia Brazil

**Keywords:** Computational science, Viral infection

## Abstract

Zika virus was responsible for the microcephaly epidemic in Brazil which began in October 2015 and brought great challenges to the scientific community and health professionals in terms of diagnosis and classification. Due to the difficulties in correctly identifying Zika cases, it is necessary to develop an automatic procedure to classify the probability of a CZS case from the clinical data. This work presents a machine learning algorithm capable of achieving this from structured and unstructured available data. The proposed algorithm reached 83% accuracy with textual information in medical records and image reports and 76% accuracy in classifying data without textual information. Therefore, the proposed algorithm has the potential to classify CZS cases in order to clarify the real effects of this epidemic, as well as to contribute to health surveillance in monitoring possible future epidemics.

## Introduction

The Zika virus (ZIKV) related microcephaly epidemic that occurred in several cities of Brazil starting in October 2015^1–3^, posed a series of challenges for health managers and the scientific community. This includes case classification and diagnosis certainty for the clinical spectrum of Congenital Zika Syndrome (CZS)^4,5^. As part of the emergency response to the Zika outbreak, the Brazilian government started an epidemiological investigation and immediately adapted the Public Health Events Register (RESP), an electronic form in which data were recorded of all children born with a reduced head circumference, according to the criteria initially established by the Ministry of Health^6^ (Brasil, 2015).

Although at the beginning of the epidemic the health services network did not have specific diagnostic tests for ZIKV in newborns, the clinical observations of cases with head circumference below the reference value were revealing. CZS had characteristics different from those produced by toxoplasmosis, others (syphilis, varicella-zoster, parvovirus B19), rubella, cyto megalovirus,and herpes (TORCH infections). It was therefore possible to establish a classification based on clinical-epidemiological criteria. This initiative was essential to determine the magnitude of the epidemic, its geographical distribution, the clinical spectrum of the syndrome, as well as to plan the health care needs of the affected children. Up until August 2017, 14,951 notifications of suspected cases of CZS had been registered in the RESP. However, about 33.7% of them still have no final classification, mainly due to the insufficient number of specialists in the health services network who are able to discuss and apply all the complex classification criteria to each case.

The paucity of experts and the large number of registered cases in RESP of CZS have increased the demand for decision support systems that can classify CZS without the need for human expertise. Machine Learning (ML) techniques can be used to create computer systems that learn through training to be able to make decisions with the smallest error possible. The focus of expert systems based on ML is not to identify the rules made by human specialists, but to obtain a decision as close as possible to that of a human specialists. For this purpose, the learning algorithms usually use simple and complex relations like conditional and nonlinear of the predictors to reach their decision. ML is a branch of artificial intelligence that employs a variety of statistical, probabilistic and optimisation techniques that allow computers to “learn” from past examples and to detect hard-to-discern patterns from data. In spite of the fact that ML techniques often generate models that are difficult to interpret, several health fields have already benefited from this approach such as cancer identification^7,8^, diagnosis by medical image^9,10^, epidemiology^11^, genetics^12^, medical diagnostic^13^, among others.

Considering that a team of specialists from different areas of health manually classified 1501 cases of CZS reported in the RESP by February 26, 2016, our objective was the development of ML algorithms to classify suspected cases of CZS from that database, using as a gold standard the classification developed by these specialists.

## Results

From September 2015 to February 2016, health professional reported 5,909 suspected CZS cases to MOH of which 1501 were selected for in depth review conducted by health specialists. Detailed descriptions of these patients were previously published^14^. In summary, the median age of the newborns was 8 days (IQR 1–57), 59.4% were female. Information about birth indicates that newborns weighed in median 2.740 kg (IQR 2.445–3.025), were born at 39 weeks (IQR 38–40) to young females (24 years IQR 20–29). Data about laboratorial tests for syphilis, HIV, toxoplasmosis, hepatitis, rubella, cytomegalovirus, herpes, dengue, chikungunya and Zika carried out on mothers or the newborns are also available; most have a higher proportion (> 60%) of missing values. From 1501 samples, 8.0% (120 cases) was classified deterministically by rule, 18.1% (272 cases) do not have text data as part to Group 1, and 73.9% (1109 cases) belong to Group 2 with text data.

### Group 1

Group 1 is composed by newborns for whom the corresponding files do not have text data. This group was classified only as discarded and somewhat probable cases. The evaluation results for each algorithm after choosing the best hyper-parameter is shown in Table 1. We choose a Random forest (RF) algorithm with 100 trees, max depth 5, and min samples split 40 for its large average accuracy in tenfold cross validation evaluation for this group. On the test group, this model had an accuracy of 76%. The performance of the chosen algorithm in a test group for each class is described in Table 2. The discarded class had a high recall rate (91%) showing that few cases that was a discarded will not be classified as discarded. However, this class showed reasonable precision (76%) in that some cases that were classified as discarded were in fact somewhat probable. Table 3 shows the confusion matrix of the RF classifier for group 1, showing that most of the discarded cases were correctly classified. Regarding the somewhat probable group, it presented reasonable precision (77%) showing that most of the cases classified as somewhat probable were correctly classified. However, there was a very bad recall result (50%) showing that half of the cases that were to be classified as somewhat probable had been wrongly classified.

**Table 1:** Comparison of models by tenfold cross validation accuracy for the diagnosis of newborns. The data set was divided according to whether the patient files do not have text data (Group 1) or have text data (Group 2). The best model for each group is indicated by bold letters. The abbreviations refer to the ML algorithm used: KNN is the k-nearest neighbor, CART is the classification and regression trees, RF is the random forest, and GB is gradient boosting.

Group 1				
Classifiers	Average (%)	SD (%)	Min (%)	Max (%)
KNN	77.013	9.218	59.091	90.909
CART	81.623	5.258	77.273	90.909
**RF**	**83.441**	**5.402**	**77.273**	**90.909**
AdaBoost	81.645	8.537	63.636	90.476
GB	81.645	8.776	63.636	90.909
**Group 2**				
KNN	75.884	3.688	70.786	82.954
CART	80.840	4.275	73.033	86.516
RF	83.324	2.698	79.775	88.636
AdaBoost	79.588	2.378	76.136	83.146
**GB**	**84.560**	**2.438**	**80.898**	**87.640**

**Table 2:** Evaluation for the best model in untrained data (test data) for each group. Group 1 is made up of patients without text data, and Group 2 patients with text data.

	Support (%)	Precision	Recall	F1-score
**Group 1**				
RF accuracy score (76.36%)				
Discarded cases	35 (63.6%)	0.76	0.91	0.83
Somewhat probable	20 (36.4%)	0.77	0.50	0.61
**Group 2**				
GB accuracy score (83.33%)				
Discarded cases	135 (60.9%)	0.91	0.91	0.91
Somewhat probable	40 (18.0%)	0.70	0.53	0.60
Moderately probable	36 (16.2%)	0.76	0.86	0.81
Highly probable	11 (4.9%)	0.56	0.82	0.67

**Table 3:** Confusion matrix for Group 1 classifications in untrained data.

N = 55	Discarded cases	Somewhat probable
Predict discarded	**32**	10
Predict somewhat probable	3	**10**

**Table 4:** Confusion matrix for Group 2 classifications in untrained data.

N = 222	Discarded cases	Somewhat probable	Moderately probable	Highly probable
Predict discarded	**123**	6	2	4
Predict somewhat probable	10	**21**	7	2
Predict moderately probable	2	2	**31**	1
Predict highly probable	0	1	1	**9**

### Group 2

**Figure 1:**
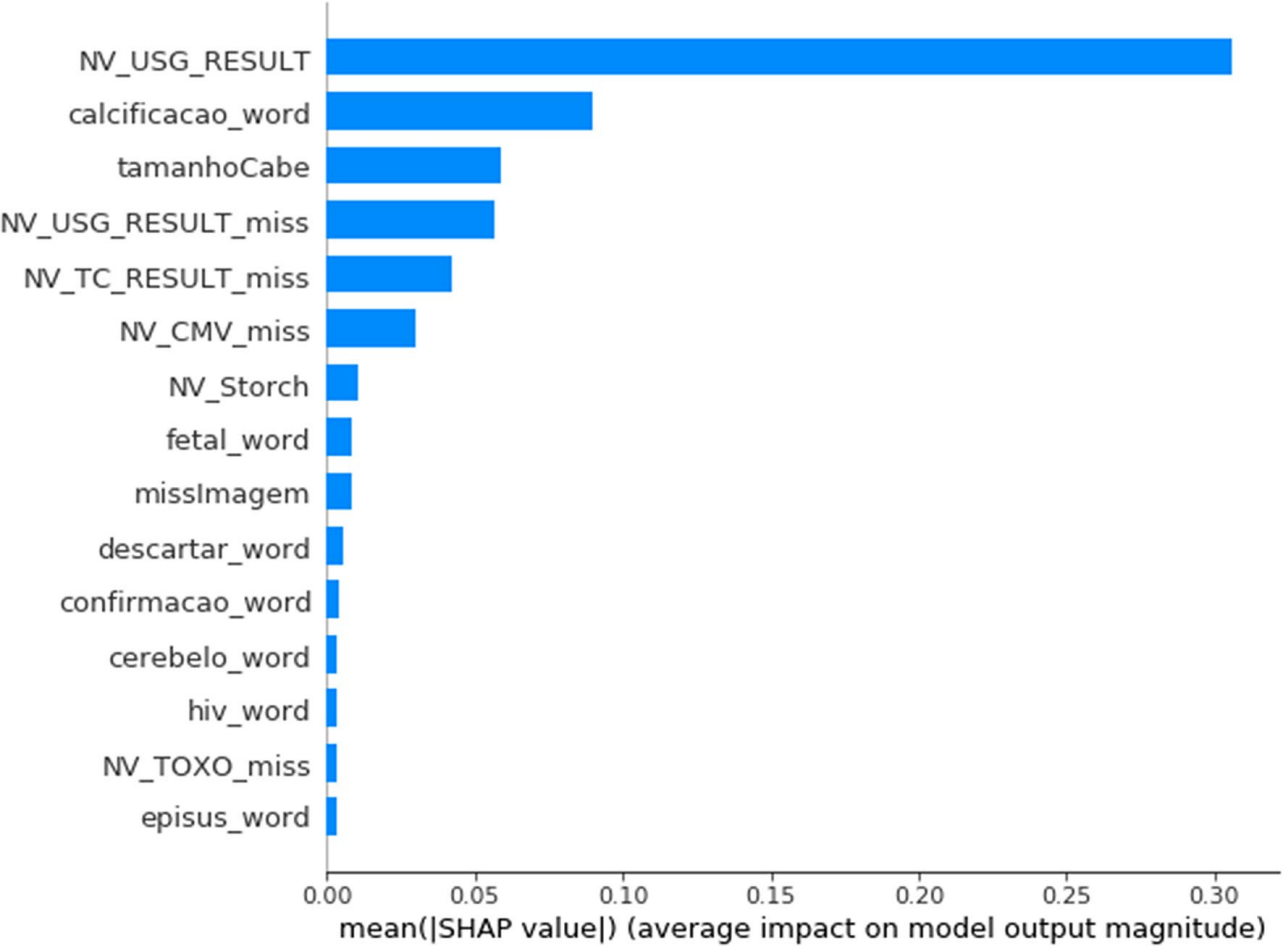
Top features in level of importance for group 2.

Group 2 is composed of newborns for whom the corresponding files included textual information. The algorithm that obtained the best accuracy based on tenfold cross validation was Gradient Boosting with max depth 8 and min samples split 5. The accuracy of this algorithm in the test group was 83%. Table 2 shows the classifier’s performance on data not previously shown to the algorithm. The Discarded class reached 91% precision, 91% recall and 91% F1 score, indicating that the algorithm was able to distinguish discarded cases from other cases well. Table 4 shows the confusion matrix for cases not presented to the classifier. It can be seen that for all classes most cases were correctly predicted. Figure 1 shows the most important features for classification. We can observe that the presence of the word calcification in the examinations and abnormal ultrasound results were an important criteria for classifying the cases of CZS.

The criteria adopted by health ministry specialists for classifying cases of CZS changed during the epidemic. The classification adopted in this work is more informative and when compared with the classification of the ministry of health we observed that both during the epidemic in 2015 and at the end in 2017 (Supplementary Material S2) the algorithm correctly classify most of the cases discarded. However, in 2017 there are more cases of discarded in the other categories, which can be explained by the change of the confirmation criteria and partly by the smaller number of cases of CZS.

## Discussion

The emergence and expansion of data from electronic medical records has the potential to revolutionize our understanding of pathology. However, access to this data may be limited by ethical concerns^15,16^ and large quantities of unstructured data as text. Automated knowledge extraction of unstructured information is not easy, for this reason, some methodologies have been proposed^17–19^. Clinical notes are usually short with many abbreviations, technical terms, changing style from doctor to doctor and, in some cases, using phrases without any formal sentence structure in the native language. Due to these difficulties, we chose to use a method that values the detection of important terms to characterize the pathology. Despite the difficulties, the use of unstructured data such as texts from medical records in conjunction with structured data has helped in classifying problems^20^. In this work, the use of text data in conjunction with structured data showed a better ability to distinguish CZS. Group 2, which contains textual data, despite having more classes to discern, showed better levels of accuracy than Group 1. When we removed the textual information from Group 2 and performed new training and assessment, the algorithm showed worse estimates.

The identification of CZS was carried out by specialists analyzing each case, as reported in previous works^14,21–23^. Due to the limited number of specialists at the time to carry out these evaluations, it is very difficult to understand the real magnitude of the frequency of CZS and obtain a sufficient amount of data to better understand which factors influence CZS.

The present algorithm showed a good ability to correctly classify the incidence of CZS. A characteristic which increases its usefulness is its ability to distinguish between discarded cases from cases with different levels of CZS probability. Since discarded cases are the vast majority of cases, this algorithm has the potential to be a useful tool for surveillance to trial probable CZS cases. The most important attributes selected by ML for the identification of CZS are the presence of neuroimaging calcifications, STORCH positivity and the measurement of head circumference in relation to the expected measurement in population. Although these characteristics are the most evident and useful to characterize the pathology, what defines each case is the presence of several other characteristics that, together with those described above, can reinforce or discard CZS. This diversity of characteristics for classification makes it difficult for experts to create simple rules and justifies the use of ML to try to replicate their decision.

The methods that obtained the best accuracy in classifying both patients with text data and those without were RF and GB, which are Ensemble Methods. This is possibly due to the composition of different models, which makes it possible to consider a great diversity of different characteristics in the classifications.

The development of this ML procedure for automatic determination of CSZ cases helps to better understand the impact of the Zika epidemic. It also has the potential to contribute to several other works for a better understanding of this syndrome as well as the monitoring of those affected to identify unobserved CZS effects.

## Methods

### Data-set and data acquisition

The data used for training and evaluating the algorithm was reported in a previous study^14^ which provided a classification scheme for CZS performed by specialists in their respective fields. Briefly, 1501 live newborns suspected of microcephaly caused by infectious diseases during pregnancy were selected in 2015. Anthropometry, serology and neuroimaging information from Public Health Event Registry (RESP) and National Birth Registration System (SINASC) were used to classify the cases into 5 categories based on the plausibility of microcephaly caused by Zika: definite cases, highly probable cases, moderately probable cases, somewhat probable cases, discarded cases.

This dataset, which is used here as a golden standard for CZS classification, was built through the linkage of the RESP and SINASC comprising the following birth related information: gestational age, head circumference and gender; serology for different infections such as Zika, dengue, chikungunya, cytomegalovirus, syphilis and toxoplasmosis; textual information from ultrasound, tomography and magnetic resonance imaging of the cranium. This dataset provide text information from the examinations, including reports, descriptions and other possible observations. Microcephaly was considered in newborns with a head circumference smaller than the 95% CI determined according to InterGrowth standards^24^, based on their respective gender and gestational age.

### Data preprocessing

138 newborns (9.2%) with missing values for gender or gestational age were considered missing for microcephaly. For all non-textual database variables with missing values, a new binary variable was generated, for which 1 represents missing values and 0 present values. Then, in original variables the missing values were input replacing the missing values with the most frequent class in categorical variables. Head circumference that had 84 (5.6%) missing values was replaced by the value 32cm. All textual variables were collapsed into a single string. First we apply a cleanup by removing common writes errors. Then the python package *spacy* version 2.2.3 with *pt_core_news_sm model* was applied to text processing as: tokenizer, tagger for detect grammatical entities, parse to recognize synthetic relationships. Then we use words attributes and relationships to identify whether words are being affirmed or denied according to context. Then remove accents, replace similar words with the same synonym and put all verbs in the same tense. An important step was to remove stop words that do not contain important information for the meaning of the sentences (for examples the words *“the”*,*“a”*,*“to”*). Besides using the stop words contained in the *pt_core_news_sm model* we added 546 new stopwords that can be found in Supplementary Material (S1). In the end, 629 different words with similar meanings were mapped to the same meanings, reducing the search space for the classifier. These words with their respective meanings can be found in the Supplementary Material (S2). Bag of words was applied with presence with denied meaning as 0, the presence and absence of each word 1 and presence with affirmative meaning as 2. This choice not count the terms was taken because most of the texts are short and well condensed in terms of information and abbreviations. For accuracy assessment, 146 words were obtained (disregarding newborns in the test group) for evaluation and a total of 171 words for final classification.

### Model training and evaluation

**Figure 2:**
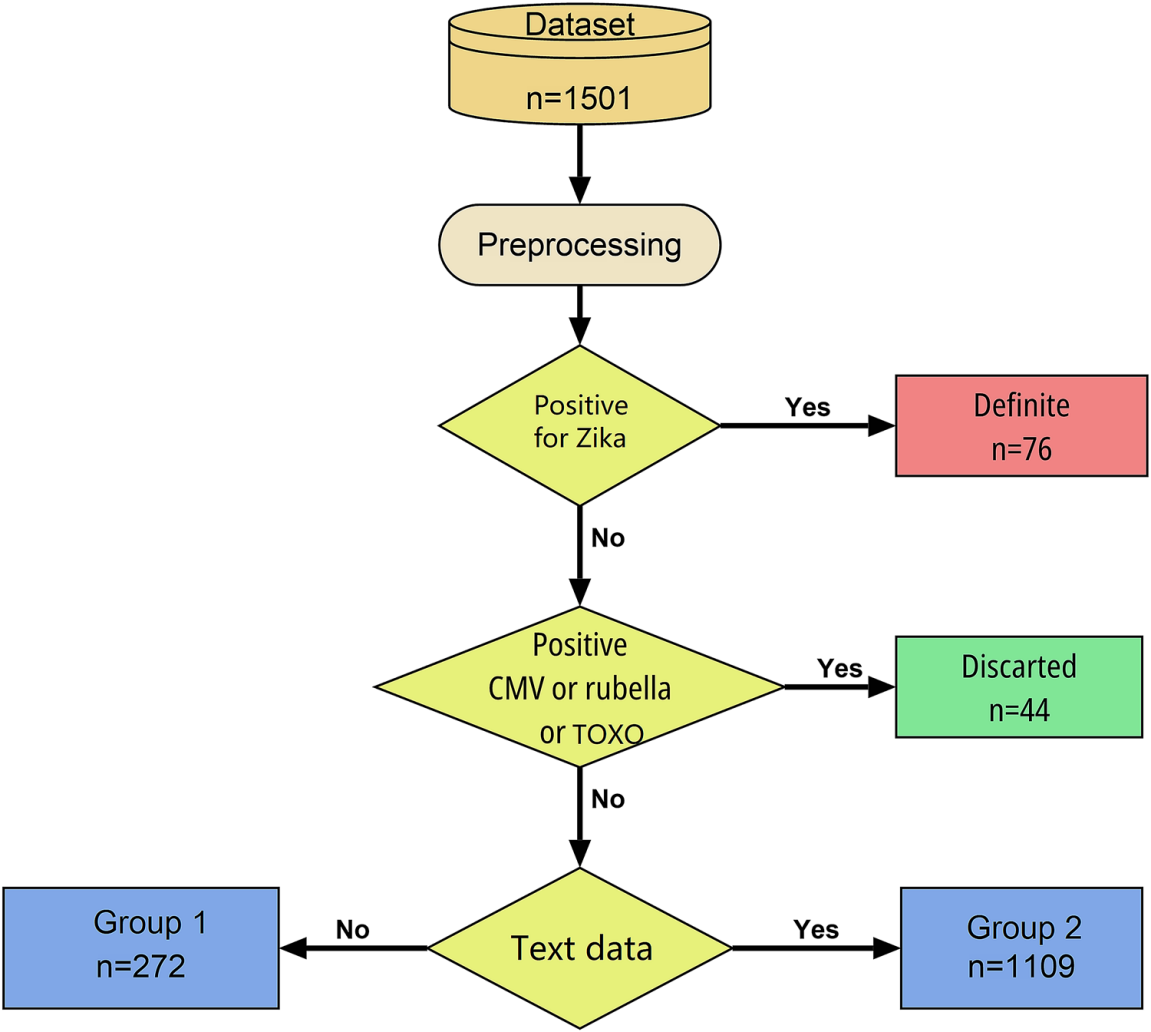
Flowchart describing the classification process, where CMV is positive serology for cytomegalovirus and TOXO is positive serology for *Toxoplasma gondii*.

The classification methodology is represented by the flowchart in Figure 2. Newborns who were positive for Zika serology were considered definitive cases for CZS. Cytomegalovirus, rubella and toxoplasmosis may also cause malformation when the fetus is affected. Therefore, when the newborn has serology positive for these diseases and negative for Zika, we classify it as a discarded case for CZS. From 1501 newborns, 120 were classified deterministically by the above rules.

The newborns, which cannot be classified as a rule, were divided into two subgroups, Group 1, formed by those without textual information (n = 272), and Group 2, formed by those with textual information (n = 1109). Classifier algorithms were applied to each group. This decision to divide into two groups was taken because the quantity and quality of the information in these two groups are very discrepant. The group that did not have textual information could only be classified by the experts as discarded cases and somewhat probable cases, because it is not possible to obtain an adequate confirmation for CZS.

The final assessment of the accuracy of the classifier was performed using a separate 20% test group. The remainder sample was used to choose an algorithm and hyper-parameters through tenfold stratified cross validation. The best hyper-parameters were chosen by grid search. The algorithms evaluated were: k-nearest neighbor (KNN), Classification and Regression Trees (CART), Random forest (RF), AdaBoost and Gradient boosting (GB). Finally, the test group was used to evaluate the performance of the chosen models. The final developed algorithm was then generated by applying the chosen models previously evaluated with their respective hyper-parameters to the total sample (n = 1501).

The final algorithm trained using all the cases classifying by rule (120 cases), group 1 (272 cases) and group 2 (1,109 cases) and was able to replicate the experts’ classification with 97% accuracy.

### Accession codes

The algorithm can be accessed at link: https://github.com/rafael-veiga/Classification-algorithm-of-Congenital-Zika-Syndrome-characterizations-diagnosis-and-validation.

## Supplementary Information

Supplementary Information.

## Data Availability

The data from the work cannot be made available for ethical reasons because it is medical records with individualized text data.
