# Supplementary material for: Classification algorithm for congenital Zika Syndrome: characterizations, diagnosis and validation
Source: Sci Rep. 2021 Mar 24;11:6770. doi: 10.1038/s41598-021-86361-5 (PMC7990918; doi:10.1038/s41598-021-86361-5)
Supplement: Supplementary file 1 — Supplementary Information. [file 41598_2021_86361_MOESM1_ESM.pdf]

Supplementary information for:

“Classification algorithm for Congenital Zika Syndrome: characterizations, diagnosis and validation”

Rafael V. Veiga, Lavinia Schuler-Faccini, Giovanny V. A. França, Roberto F. S. Andrade, Maria Glória Teixeira, Larissa C. Costa, Enny S. Paixão, Maria da Conceição N. Costa, Maurício L. Barreto, Juliane F. Oliveira, Wanderson K. Oliveira, Luciana L. Cardim, and Moreno S. Rodrigues

*rafaelvalenteveiga@gmail.com

Supplementary Material (S1): Stop words list

06h	07h	0a	10dias	10ui	110x70
12d	12s	13s	14sm	15sm	17h
18s	1dia	1gg	1gm	1odose	1ot
200ui	200x190	20150vdrl	20mcg	20s	20s6d
21dp	21sm	22s	23s	23sm	24h
25s	26semanas	28sem	2d	2dp	2sm
2tri	30s	31s	33sem	34s	36s
36s3d	36sm	37s1d	37s2d	37s5d	38oc
38s	38s	38s1d	38sem	38semanas	39s
39sm	3omes	3tri	48h	4sm	51ui
5ag	6consultas	6d	9797ba	a	a0
a1	aas	ab	abar	abd	abril
absag	ac	aceito	achar	acirc	acm
acolhido	acolhimento	acometer	acompanhamento	acompanhante	acompanhar
adequadamente	adequar	adicionar	adm	adm	administrar
admissao	admissional	admitir	adquirir	af	afirmar
ag	agendar	agente	aghabs	aguardar	aig
aina	alagoinhas	albert	algum	alignjustify	alves
am	ambigua	amostrar	analisar	andamento	anit
ano	anotacao	aometodo	ap	aparentar	aparente
apo	apr	aprese	apresentando	apresentar	aproximadamente
arlinda	aspecto	aspectos	ass	assistencia	assistente
associado	associar	assumir	at	atar	atb
atendido	atendimento	ater	atrasar	atravez	atual
atualizacao	atualizado	aume	aus	ausentar	automedicacao
av	avaliacao	avaliar	b	bebr	biometria
bo	born	br	buscar	ca27	ca28
caber	caetano	caf	camacari	capibarib	captar
caracteristico	caracteriza-se	caracterizacao	caracterizar	caratcerizada	caribe
carlos	cartao	carteiro	casar	caso	causar
chegar	chorar	cid	cidade	cigarro	citar
civil	clareza	classificacao	classificar	clinicamente	clinicar
clinico	cm	coeficiente	coleta	coletadas	coletado
coletados	coletou	colher	colhido	comentario	comer
comp	companheiro	comparecer	compativeis	compativel	compensatoria
compensatorio	completar	comprimento	computar	comunicar	conhecer
conjugal	conjuge	conseguir	considerar	consumir	contar
continuar	correspondente	corresponder	d	datada	derecionado
descobrir	descrever	descrito	devidamente	dezembro	dia
dia018	dia16	diariamente	dimens	dimensao	dirigir
div	documento	dosar	dose	doutor	dr
duarte	durante	durar	duvidar	e	efeito
elisa	email	encaminh	encaminhamento	encaminhar	encontrar
enfermeiro	engravidar	ensinar	entanto	enviar	episodio
epoca	equipar	esclarecer	escolaridade	espacar	especialmente
especificacao	especificar	esperado	esperar	esquema	estudar
estudo	etc	eunapolis	evidenciar	evidente	exam
exame	expediente	favorecer	fem	feminino	ficar
filho	formular	freitas	fundamental	gilmara	gravidez
gravido	gusmao	havia	hipotese	histori	historia
historiar	historico	hoje	horar	hospital	hospitalar
hospitalizacao	id	idade	identificar	ig18	ig37
ig9	ignorar	igual	imagem	impressao	indicar
indice	infectologista	informacao	informar	ingerir	instituicao
instituto	internacao	internamento	internar	investigacao	investigar
irar	irm	irma	irmao	itabaiana	jaguare
janeiro	janine	joao	jose	josy	julho
juliete	junho	juntamente	kg	l	laboartoriais
laboratorial	laboratorias	laboratorio	lacen	lactente	laudo
lauro	lembrar	levantar	levar	like	m

m30	macho	mail	maio	major	mamao
maranhao	marco	masculino	materni	maternidade	materno
medir	meireles	mencao	mensurar	merecer	mg
mim	ml	mm3	mmhg	moderar	morador
mostrar	municipal	municipio	municpal	mutirao	nome
notadamente	notar	notfic	notificacao	notificar	noto
novembro	nph	o	o80	obs	observacao
observam-se	observar	obter	obtido	ocorrer	odor
ofertar	ola	osvaldo	outro	outubro	p
p0	p50	p5915	p706	pa	paciente
pai	paiente	pantanal	paraiba	parceiro	parecer
patente	paulo	pc	pedir	pedriatras	perceber
perceptivel	pereira	periodo	permitir	pescador	pesquisar
pessoa	pessoal	planilha	podendo	policia	possibilidade
preescrito	preferir	presidiario	primar	profissional	prontuario
prontuarios	protocolo	protuario	provavel	provavelmente	quaestionario
quantidade	questionario	realata	receber	recife	recordar
registrar	registro	relacionado	relacionar	relatar	relato
relatorio	representar	resultar	revelar	revisao	roberto
sair	sala	salvador	samara	santo	secretariar
secundario	seguinte	seguir	semana	semanal	semanss
semestre	servico	setembro	silvar	silviano	situacao
sobretudo	soicitado	solange	soliciotado	solicitar	solicitodo
solteiro	souza	sr	stylefont	substanciar	sugerimos
sugerindo	sugerir	sugestivo	sul	sumario	tecnica
tecnicamente	tecnico	tel	telefonar	telefonico	telefonicos
testar	tio	tornar	trabalhar	transcorrer	transcrever
transferir	tratamento	tratar	trimestre	ultima	umar
unica	unico	unidade	universitario	usuaria	usuario
utilizar	v	valenca	vestir	viagem	vigilancia
vir	visibilizacao	visitar	visto	visualizar	voluntario

Supplementary Material (S2): Similar words list

atild:atilde
antihiv:hiv
anti:anticorpo
ant:anterior
anomalidade:anormal
ambulatorio:ambulatorial
abdomen:abdome
acentuacao:acentuada
acidar:acido
agravar:severo
alcoolicas:alcoolismo
aumento:crescimento
alto:acentuada
anormais:anormal
assimetrico:assimetria
alteracoes:alteracao
artralgia:artropatia
adelgacamento:reducao
bebido:beber
baar:ebv
branca:branco
curto:discreto
contatos:contato
congenitas:congenita
colecao:variadas
citomrgalovirus:citomegalovirus
citomagalovirus:citomegalovirus
chuikungunya:chikungunya
chikun:chikungunya
cesareo:cesarea
cefalicac:cefalico
cardiopatia:cardiaco
calcificacoesreducao:calcificacao
calc:calcificacao
calcificacoes:calcificacao
capsulo:capsula
cerebrais:cerebro
cistica:cisto
compensataria:compensatoria
cornar:corneo
cortical:cortex
craniano:cranio
disturbios:anormal
dignosticada:confirmacao
diagnosticar:diagnostico
detectavel:deteccao
desaparecer:reducao
deformidade:anormal
dandy:dandy-walker
definido:confirmacao
diametros:circunferencia
dilatar:crescimento
diminuicao:reducao
distribuidas:distribuida
dilatacao:crescimento
exatema:exantema
exantematica:exantema
estabelecido:confirmacao
epstein:ebv
ecocardioigrama:ecocardiograma
elevar:acentuada
esquer:esquerdo
esparsas:discreto
fumar:tabaco
feto1:fetal
falha:anormal
feto:fetal
glicose:glicemia
gestacional:gestacao
gest:gestacao
hsag:hbs
hipoplasicas:hipoplasico
hiperextensao:crescimento
hepb:hbs
hemmorragia:hemorragia
hbshg:hbs
habitual:comumente
hidranencefalia:hidrocefalia
holoprosecefalia:holoprosencefalia
intrauterinas:intrauterino
intercorrencias:intercorrencia
insuficiencia:reducao
incomple:incompleto
ilicitas:ilicito
icterico:ictericia
inexpecifica:inespecifico
irregular:anormal
linf:linfocitos
lesao:anormal
later:lateral
lisencefalicas:lisencefalia
localizar:localizadas
microoftalmia:microftalmia
micro:microcefalia
medicar:medicamentar
mediar:medicacao
malfor:malformacao
macrocalcificacoes:calcificacao
morfas:morfologico
multiplo:variadas
norm:negativo
apoptose:reducao
antihcv:hcv
anteriormente:anterior
anencefalia:anencefalo
anomalia:anormal
altracoe:anormal
abdominal:abdome
acentuar:acentuada
administracao:administrar
alteracao:anormal
alcoolico:alcoolismo
agrupar:agrupadas
altas:acentuada
arterial:arteria
assimetricos:assimetria
atrial:atrio
artrogripose:artropatia
afrofia:reducao
bebiba:beber
bilatera:bilateral
cvm:citomegalovirus
cronicas:cronica
constatar:confirmacao
cong:congenita
cmvi:citomegalovirus
citomegalovir:citomegalovirus
citomeg:citomegalovirus
chk:chikungunya
chiku:chikungunya
cerebr:cerebro
cefalica:cefalico
cardiop:cardiaco
calcificacoe:calcificacao
cafalica:cefalico
calcificar:calcificacao
centrar:centralizacao
cerebral:cerebro
cisticas:cisto
conclusao:confirmacao
corno:corneo
corticos:cortex
duplicidade:duplo
disponiveis:disponivel
diferente:anormal
diagnostica:diagnostico
detectar:deteccao
dengu:dengue
deformar:anormal
dondy:dandy-walker
destaca:acentuada
difusamente:difusa
dilatacao:crescimento
discreta:discreto
distribuir:distribuida
diminuicao:reducao
exantematima:exantema
evidencia:confirmacao
espont:espontaneo
encefalico:encefalo
ecocardiog:ecocardiograma
encefalica:encefalico
encefal:encefalo
esquerda:esquerdo
fumante:tabaco
febril:febre
fechamento:fechar
fissurar:fissuras
glic:glicemia
gestaca:gestacao
giros:giro
hivs:hiv
hipertesnao:hipertensao
hiperestensao:crescimento
hepatitec:hcv
hemoglobia:hemoglobina
hbsg:hbs
hemisferios:hemisferio
hiperecoicos:hiperecoicas
irritabilida:irritabilidade
intrauterina:intrauterino
intercorr:intercorrencia
infec:infeccao
incomp:incompleto
igm0:igm
involucao:reducao
infecciosa:infeccao
interior:interno
leuco:leucocito
lacerar:anormal
lados:lateral
linha:linear
morfologico:morfologia
microcefafia:microcefalia
micricefalia:microcefalia
medicamento:medicamentar
mediante:medicacao
malfomalcao:malformacao
microcalcificacoes:calcificacao
morfologica:morfologico
normalidade:negativo
nervosoatipico:nervoso
antihvc:hcv
aghbs:hbs
anteceder:anterior
anamalia:anormal
amni:amniotico
altercoes:anormal
abortar:aborto
acentuda:acentuada
adoecimento:doenca
alcool:alcoolismo
alcoollica:alcoolismo
alargamento:crescimento
amniotica:amniotico
arterias:arteria
atrofia:reducao
atrofico:reducao
articular:articulacao
bpm:bcf
barriga:abdome
bilaterais:bilateral
cutis:cutaneo
cranio:cefalico
consangu:consanguinidade
confirmatorio:confirmacao
cmvconsta:citomegalovirus
citomegalovi:citomegalovirus
circunfer:circunferencia
chikungunia:chikungunya
chik:chikungunya
centralizar:centralizacao
cefalecina:cefalexina
cardiaca:cardiaco
calcificaco:calcificacao
cabeca:cefalico
calsificacao:calcificacao
cerebelar:cerebelo
cerebra:cerebro
cistico:cisto
confirmada:confirmacao
cornos:corneo
corporal:corpo
doer:dor
dilat:crescimento
diarreico:diarreia
diagnostic:diagnostico
detec:deteccao
deng:dengue
deficit:reducao
walker:dandy-walker
determinando:confirmacao
difusas:difusa
dimensoes:dimensao
discretamente:discreto
delgado:discreto
disproporcao:anormal
exantematico:exantema
etiologia:etiologico
espalhar:esparso
ecograficos:ecografico
ecocardiagrama:ecocardiograma
encefalo:encefalico
encefalico:encefalo
esquerdar:esquerdo
fraco:discreto
febrel:febre
fusao:fechar
focos:foco
gicemia:glicemia
gestac:gestacao
giral:giro
hipoxico:hipoxia
hipertenso:hipertensao
heterogenea:heterogeneo
hepatiteb:hbs
hematrocrito:hematocrito
hbsag:hbs
hemorragico:hemorragia
hipodensa:hipodensidade
irradiar:esparso
intracranianas:intracraniana
intenso:severo
inespecificas:inespecifico
inchaco:crescimento
igg10:igg
inespecfico:inespecifico
infeccioso:infeccao
isquemica:isquemico
leucocitos18:leucocito
laterais:lateral
lineares:linear
lobo:lobos
mon:monocitos
microcefali:microcefalia
microcalcificacao:calcificacao
medicacoes:medicamentar
malformaca:malformacao
magnetico:rm
microcefalico:microcefalia
multiplas:variadas
normalida:negativo
nenhum:negativo
antihtlv:htlv
antihbs:hbs
antecedente:anterior
anormalidade:anormal
aminiotico:amniotico
aintihcv:hcv
abaixar:abaixo
agenesia:agnesia
afilamento:discreto
alcoolica:alcoolismo
aumentar:crescimento
alta:acentuada
anecoica:anecoico
assimetrica:assimetria
atrofiar:reducao
artra:artropatia
ausente:ausencia
bilitest:bilirrubina
baixar:reducao
bilateralmente:bilateral
cuteneo:cutaneo
crak:crack
congenito:congenita
comprobatorio:confirmacao
cmv:citomegalovirus
citomegalosvirus:citomegalovirus
circ:circunferencia
chikungunha:chikungunya
chicungunya:chikungunya
cefalicas:cefalico
carniana:cefalico
calficicacoes:calcificacao
calcifi:calcificacao
cabecas:cefalico
capsular:capsula
cerebelarcranio:cerebelo
circunvolucao:circunferencia
cistos:cisto
confirmar:confirmacao
corticais:cortex
craniana:cranio
doen:doenca
dignostico:confirmacao
diagnosticas:diagnostico
diag:diagnostico
destruicao:anormal
degeneracao:reducao
deficiencia:reducao
definidas:confirmacao
diametro:circunferencia
difuso:difusa
diminuido:reducao
dimorfismo:dismorfismo
desproporcao:anormal
excessivo:severo
exantematicas:exantema
estreito:discreto
espaco:esparso
ecografia:ecografico
ecogenicidade:ecogenico
esq:esquerdo
enchimento:crescimento
estreitamento:reducao
feto2:fetal
familiar:familia
face:facial
frontais:frontal
gestcao:gestacao
gesta:gestacao
grosseiras:grosseiro
hipotireioidismo:hipotireoidismo
hipertensivo:hipertensao
herpes:hsv
hepaptite:hepatite
hbc:hcv
hbcv:hcv
hidroanencefalia:hidrocefalia
hipoplasia:reducao
intrautero:intrauterino
intracerebral:intracraniana
insuficiente:reducao
indeterminar:inderteminado
implatacao:implantacao
identificacao:confirmacao
inespecificos:inespecifico
injuria:anormal
luxacao:crescimento
leucocitos:leucocito
lisenfalia:lisencefalia
lisencefalias:lisencefalia
lobolos:lobos
minimo:reducao
micr:microcefalia
mental:cognitivo
medicacao:medicamentar
malforma:malformacao
magnetica:rm
microcrania:microcefalia
multiplos:variadas
normal:negativo
negativar:negativo

Supplementary Material (S3): ML classification of CZS applied to confirmed and discarded cases in RESP 2015 and 2017.

RESP 2015	Confirmed (%)	Discarded (%)
Discarded	132 (6.2%)	1987 (93.8%)
Somewhat probable	292 (52.1%)	268 (47.9%)
Moderately probable	321 (85.6%)	54 (14.4%)
Highly probable	77 (86.5%)	12 (13.5%)
Definite	176 (99.4%)	1 (0.6%)
**RESP 2017**	**Confirmed (%)**	**Discarded (%)**
Discarded	37 (17.3%)	177 (82.7%)
Somewhat probable	53 (31.9%)	113 (68.1%)
Moderately probable	31 (49.2%)	32 (50.8%)
Highly probable	15 (44.1%)	19 (55.9%)
Definite	24 (70.6%)	10 (29.4%)
